# Chemical shift assignments of the C-terminal domain of CaBP1 bound to the IQ-motif of voltage-gated Ca^2+^ channel (Ca_V_1.2)

**DOI:** 10.1007/s12104-022-10108-0

**Published:** 2022-09-05

**Authors:** Ian Salveson, James B. Ames

**Affiliations:** Department of Chemistry, University of California, Davis, Davis, CA 95616 USA

**Keywords:** CaBP1, EF-hand, Ca_V_1.2, IQ-motif, Calmodulin, CDI

## Abstract

The neuronal L-type voltage-gated Ca^2+^ channel (Ca_V_1.2) interacts with Ca^2+^ binding protein 1 (CaBP1), that promotes Ca^2+^-induced channel activity. The binding of CaBP1 to the IQ-motif in Ca_V_1.2 (residues 1644–1665) blocks the binding of calmodulin and prevents Ca^2+^-dependent inactivation of Ca_V_1.2. This Ca^2+^-induced binding of CaBP1 to Ca_V_1.2 is important for modulating neuronal synaptic plasticity, which may serve a role in learning and memory. Here we report NMR assignments of the C-terminal domain of CaBP1 (residues 99–167, called CaBP1C) that contains two Ca^2+^ bound at the third and fourth EF-hands (EF3 and EF4) and is bound to the Ca_V_1.2 IQ-motif from Ca_V_1.2 (BMRB accession no. 51518).

## Biological context

Ca_V_1.2 controls the excitability of the postsynaptic membrane in hippocampal neurons, which is important for learning and memory (Hell et al. 1993; Moosmang et al. 2005; Vogl et al. 2015). The cytosolic C-terminal region of Ca_V_1.2 (residues 1644–1665, called IQ-motif) is important for promoting Ca^2+^-dependent inactivation (CDI) of Ca_V_1.2 (Erickson et al. 2001; Zuhlke et al. 1999). Ca^2+^-free CaM has been suggested to bind to the IQ-motif to increase the channel open probability under basal conditions (Adams et al. 2014; Erickson et al. 2001). Ca_V_1.2 channel opening promotes a rise in intracellular Ca^2+^ sensed by CaM that causes a Ca^2+^-induced conformational change in the CaM/Ca_V_1.2 complex leading to CDI (Ben Johny et al. 2013; Peterson et al. 1999; Zuhlke et al. 1999). CaBP1 competes with CaM for binding to the IQ-motif (Findeisen et al. 2013; Hardie and Lee 2016), which prevents channel pre-association of CaM and abolishes CDI (Hardie and Lee 2016; Oz et al. 2011). Thus, CaBP1 serves as a competitive inhibitor of CDI and promotes constitutive channel activation at high Ca^2+^ levels (Hardie and Lee 2016; Oz et al. 2011), in contrast to CaM that causes Ca^2+^-induced channel inactivation (Peterson et al. 1999; Zuhlke et al. 1999). In essence, CaBP1 and CaM oppositely regulate Ca_V_1.2 channel activity by serving as an accelerator and brake, respectively (Ames 2021). We report NMR resonance assignments for the C-terminal domain of CaBP1 (CaBP1C) with two Ca^2+^ bound that is bound to the IQ-motif of Ca_V_1.2 (called CaBP1C-IQ) as a first step toward elucidating the intermolecular contacts between CaBP1 and Ca_V_1.2.

## Methods and experiments

### *Preparation of CaBP1C bound to the Ca*_*V*_*1.2 IQ-motif*

A cDNA of *Homo sapiens* CaBP1C was subcloned into pET-11b vector (Novagen) that produced recombinant CaBP1C without any extra residues. Recombinant CaBP1C uniformly labeled with ^13^C and ^15^N was expressed in bacterial cells grown on M9 minimal media supplemented with ^15^N-labeled NH_4_Cl (1 g per liter of cell culture) and ^13^C-labeled glucose (3 g per liter). The isotopically labeled CaBP1C was purified as described previously (Li et al. 2009). A peptide representing the Ca_V_1.2 IQ-motif (residues 1642–1665) was purchased from GenScript, dissolved in DMSO-d_6_ and quantified using UV–Vis absorption. A 2.0-fold excess of IQ peptide was added to Ca^2+^-bound CaBP1C and the complex was concentrated to 500 μM in the presence of 2 mM CaCl_2_ using a 3 K Amicon concentrator.

### NMR spectroscopy

**Fig. 1:**
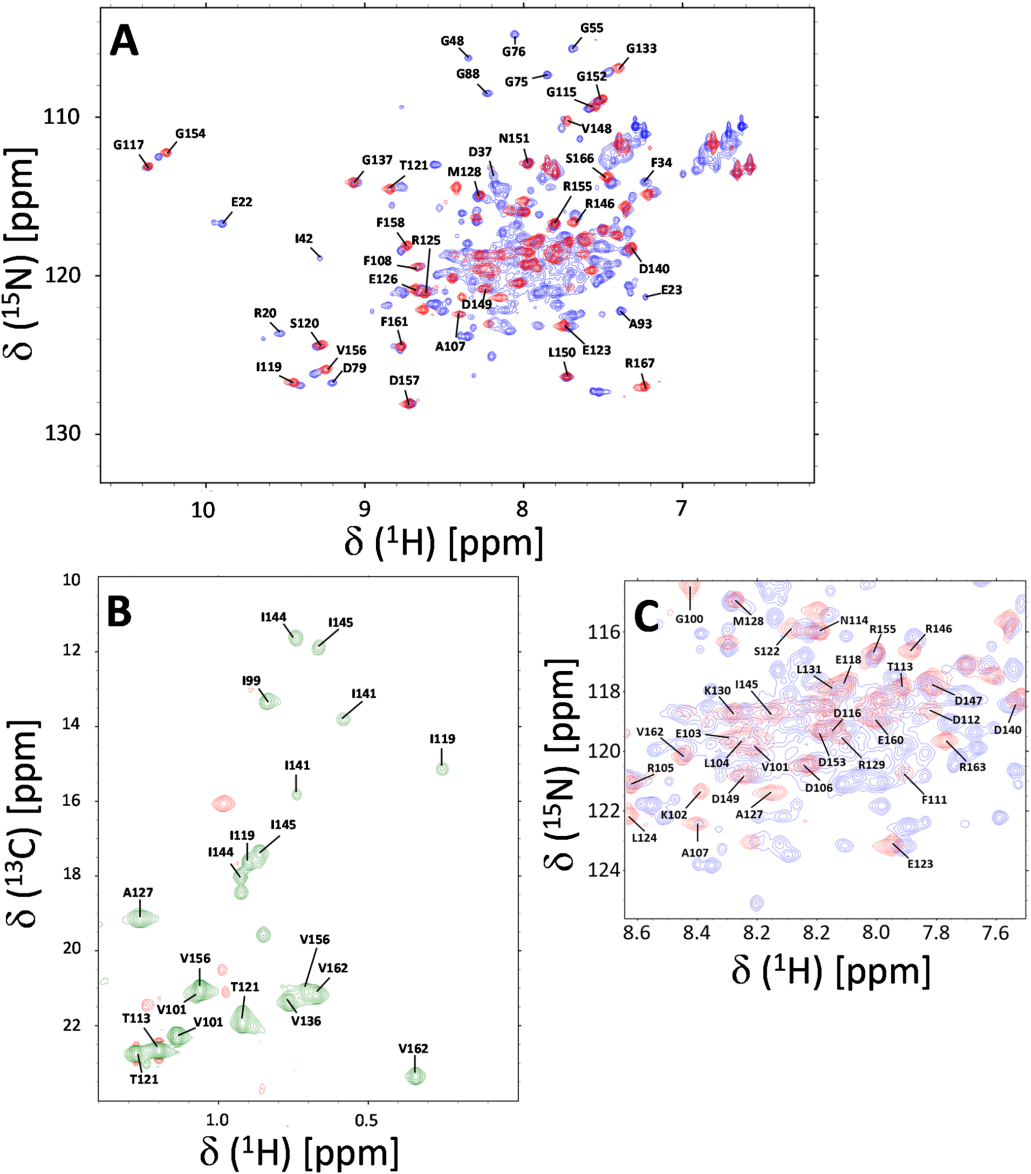
**A** Two-dimensional ^15^N-^1^H HSQC NMR spectrum of ^15^N-labeled full-length CaBP1 (blue) and CaBP1C (red) both bound to unlabeled Ca_V_1.2 IQ peptide at pH 7.5 recorded at 600-MHz ^1^H frequency. **B** Constant-time ^13^C-^1^H HSQC spectrum of ^13^C-labeled CaBP1C bound to unlabeled IQ peptide. **C** Expanded view of the spectrally crowded region from panel **A**. Representative assignments are indicated by the labeled peaks; complete assignments are available as BMRB accession no. 51518

Samples of CaBP1C-IQ for NMR were prepared by exchanging the complex into a buffer containing 20 mM Tris-d_11_ (pH 7.5) with 2 mM CaCl_2_, and 92% H_2_O/8% D_2_O. All NMR experiments were performed at 308 K on a Bruker Avance 600 MHz spectrometer equipped with a four-channel interface and triple resonance cryogenic (TCI) probe. The ^15^N-^1^H HSQC spectrum (Fig. 1) contained 256 × 2048 complex points for ^15^N(F1) and ^1^H(F2), respectively. Backbone resonances were assigned by analyzing HNCA, HNCACB, CBCA(CO)NH, HNCO (Ikura et al. 1990). Side chain resonances were assigned by analyzing HCCCONH-TOCSY, HCCH-TOCSY as described previously (Ikura et al. 1991). The NMR data were processed using NMRFx Analyst (Norris et al. 2016) and analyzed using Sparky NMRFAM (Lee et al. 2015).

## Extent of assignments and data deposition

Ideally, we would want to perform NMR structural analysis of the full-length CaBP1 bound to the Ca_V_1.2 IQ. Unfortunately, the full-length CaBP1 forms a dimer and other oligomeric species at the protein concentrations required for 3D NMR, and the relatively large size of the protein dimer reduces the sensitivity of the triple resonance 3D NMR experiments. Instead, we chose to perform NMR structural analysis of the C-terminal domain of CaBP1 (residues 99–167, called CaBP1C) that is monomeric in solution (Li et al. 2009) and exhibits functional binding to the IQ peptide (Fig. 1A). A two-dimensional ^15^N-^1^H HSQC NMR spectrum of full-length CaBP1 bound to the IQ peptide (at 0.05 mM protein concentration where CaBP1-IQ is monomeric) demonstrates that the chemical shifts of the CaBP1 C-terminal domain are similar to those of CaBP1C bound to the IQ (Fig. 1A, C). The chemical shifts of the C-terminal domain residues of CaBP1 bound to the IQ are quite different from those of CaBP1 in the absence of the IQ, demonstrating that the IQ peptide binds to the CaBP1 C-terminal domain (Wingard et al. 2005). Also, the chemical shifts of the N-terminal domain residues of CaBP1 bound to the IQ (Fig. 1A) are the same (within experimental error) as those of CaBP1 in the absence of the IQ (Wingard et al. 2005). These results demonstrate that the IQ peptide binds solely to the C-terminal domain of CaBP1 (without contacting the N-terminal domain), and IQ binding to CaBP1C is structurally similar to that of full-length CaBP1.

The two-dimensional ^15^N-^1^H HSQC NMR spectrum of ^15^N-labeled CaBP1C bound to unlabeled Ca_V_1.2 IQ peptide (called CaBP1C-IQ) illustrates representative NMR assignments (labeled red peaks in Fig. 1A). NMR assignments were derived from triple resonance NMR experiments performed on ^13^C/^15^N-labeled CaBP1C bound to unlabeled IQ peptide. The high level of chemical shift dispersion indicates that CaBP1C-IQ complex is stably folded. The large downfield chemical shifts of the amide resonances assigned to G117 and G154 confirm that Ca^2+^ is bound to EF3 and EF4 in CaBP1C (Fig. 1A). Other noteworthy downfield shifts were assigned to I119, S120 and V156 that are located in the EF-hand β-strands and are predicted to form antiparallel β-sheets with strong backbone amide hydrogen bonds. The upfield-shifted chemical shifts of methyl resonances assigned to residues I119, I141 and V162 (Fig. 1B) suggest that these residues may be located in the hydrophobic core near aromatic residues. More than 87% of the main chain ^13^C resonances (^13^Cα, ^13^Cβ, and ^13^CO), 85% of backbone amide resonances (^1^HN, ^15^N), and 74% of methyl side chain resonances were assigned. Side chain aromatic resonances from F108, F111, F158, and F161 appear exchange broadened in the NMR spectra and could not be assigned. The unassigned amide resonances from non-proline residues (110, 122, 125, 132, 134, 138, 139, 140, 141, 142, 143, 159, 164) had weak or missing NMR intensities that prevented their assignment. In particular, a stretch of residues between EF3 and EF4 (residues 138–143) could not be assigned due to weak NMR intensities, perhaps because this linker region is conformationally disordered or otherwise unstructured. A complete list of the chemical shift assignments (^1^H, ^15^N, ^13^C) of CaBP1C-IQ have been deposited in the BioMagResBank under accession number 51518.

**Fig. 2:**
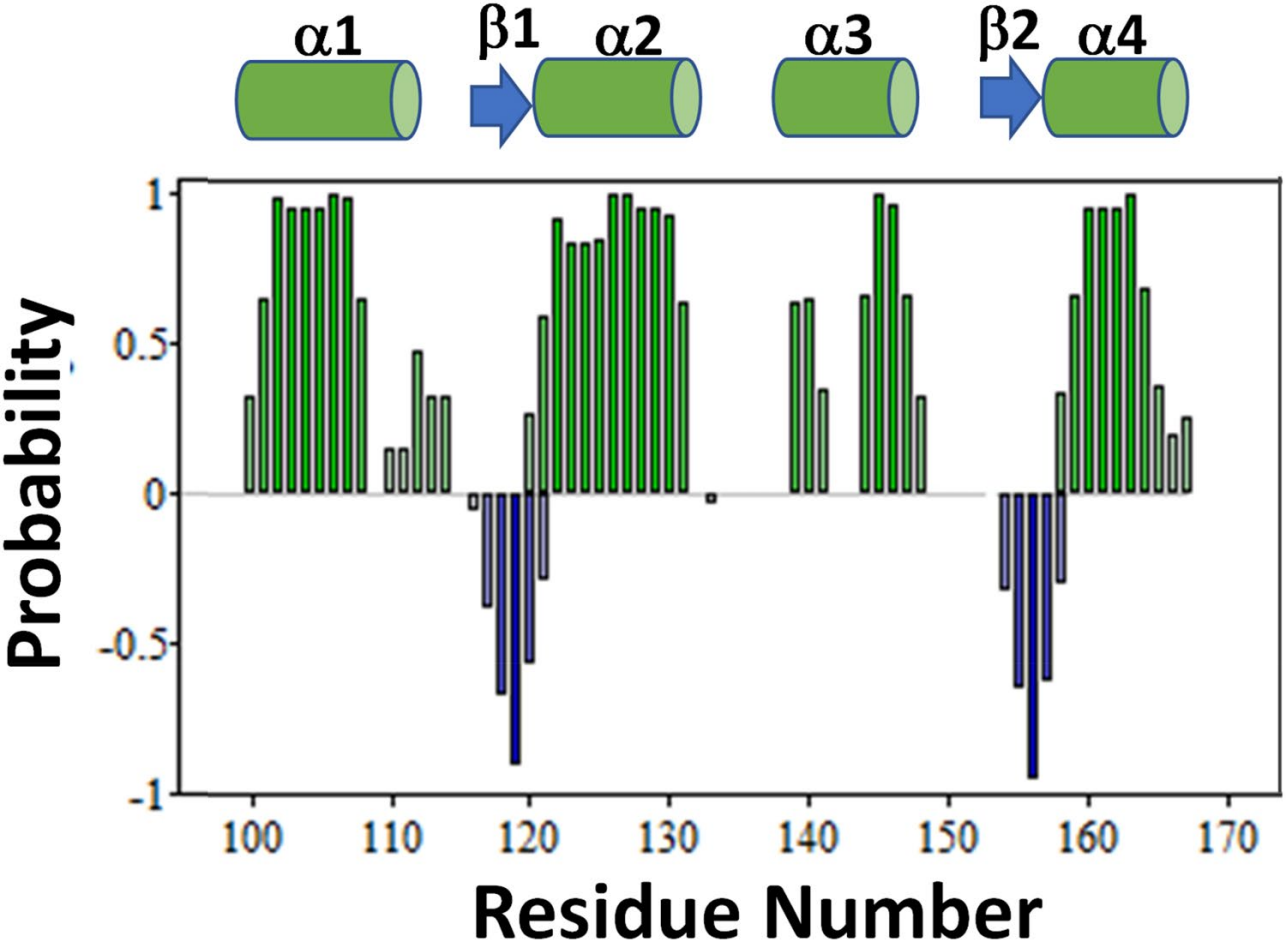
Secondary structure of CaBP1C bound to Ca_V_1.2 IQ. TALOS ANN-secondary structure probability plotted as a function of residue number. Secondary structure elements are represented as green cylinders (helix) and blue arrows (β-strand)

**Fig. 3:**
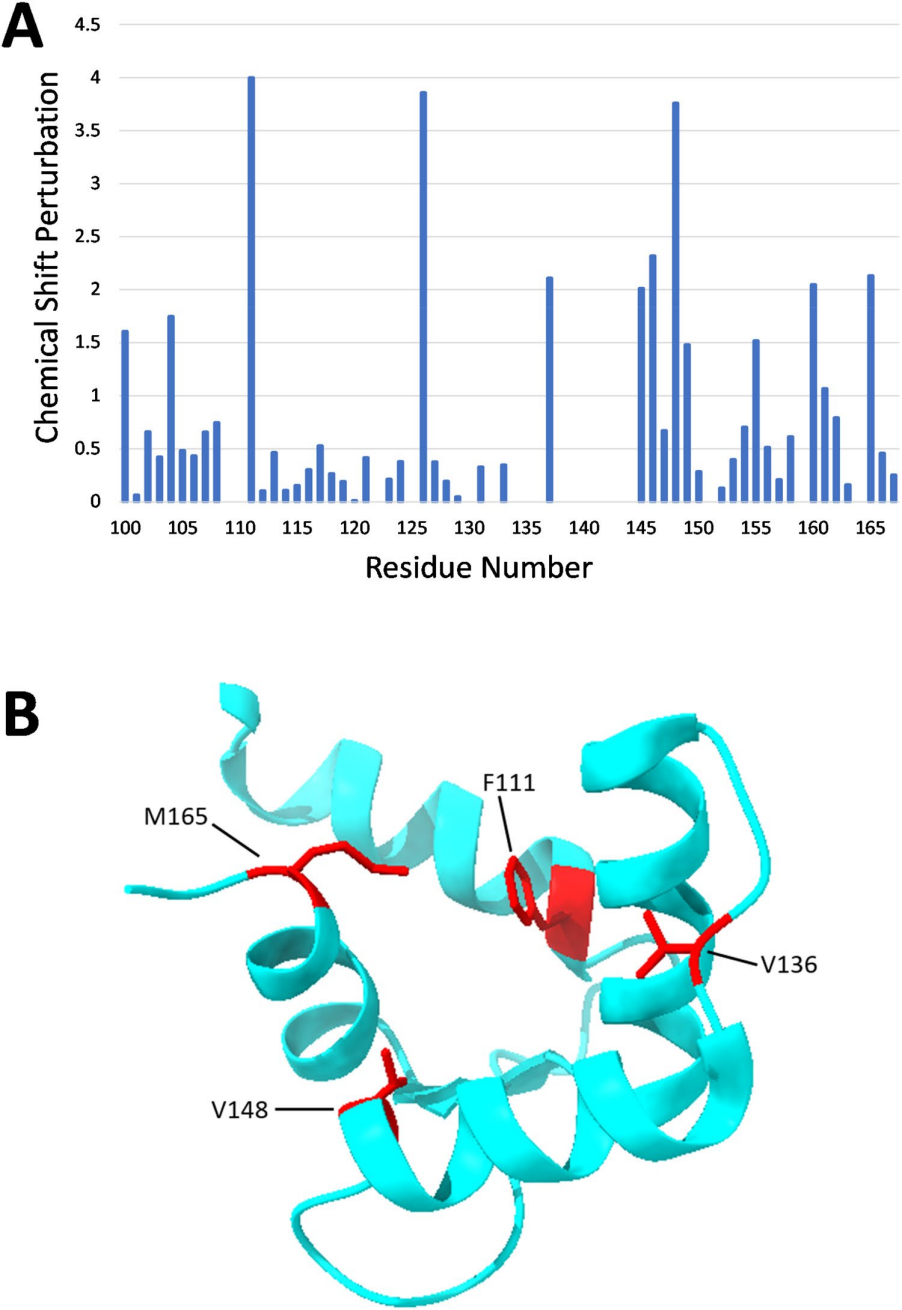
**A** Amide chemical shift perturbation for CaBP1C caused by the binding of IQ peptide. Chemical shift perturbation (CSP) was calculated as CSP = {(HN_A_–HN_B_)^2^ + (^15^N_A_–^15^N_B_)^2^}^1/2^. HN_A_ and HN_B_ are HN chemical shift of CaBP1C in the presence versus absence of IQ peptide, and ^15^N_A_ and ^15^N_B_ are ^15^N chemical shift of CaBP1C in the presence versus absence of IQ peptide. Chemical shifts of CaBP1C (in the absence of IQ) were obtained from BMRB 15623. **B** Crystal structure of the C-terminal domain of CaBP1 (Findeisen and Minor 2010). Exposed hydrophobic residues with the largest chemical shift perturbation are highlighted in red

The secondary structure of CaBP1C bound to the IQ peptide was calculated on the basis of chemical shift index (Wishart et al. 1992) and ANN-secondary structure prediction using TALOS (Shen et al. 2009) (Fig. 2). As expected, CaBP1C-IQ contains two EF-hands with four α-helices: α1 (residues 101–110), α2 (residues 121–132), α3 (residues 140–148) and α4 (residues 158–165) shown as green cylinders in Fig. 2. Conserved β-strands were observed in EF3 (residues 118–120, β1) and EF4 (residues 155–157, β2) shown as blue arrows in Fig. 2. The overall secondary structure of CaBP1C-IQ is similar to that of the crystal structure of CaBP1 (Findeisen and Minor 2010). A plot of the amide chemical shift perturbation caused by the binding of the IQ peptide reveals that CaBP1C residues F111, E126, V136, R146, V148, E160 and M165 exhibit the largest CSP values (Fig. 3A). Hydrophobic residues F111, V136, V148 and M165 are located in an exposed cleft in the crystal structure that are likely making intermolecular hydrophobic contacts with the IQ peptide (Fig. 3B). The NMR assignments of CaBP1C bound to the Ca_V_1.2 IQ-motif are a first step toward determining the three-dimensional structure of CaBP1 bound to Ca_V_1.2.

## Data Availability

The assignments have been deposited to the BMRB under the accession code: 51518.
